# Pharmaceutical Process

**Published:** 1800-08

**Authors:** 


### PHARMACEUTICAL PROCESS.

To obtain acid of tartar in a very pure ftate, Mr. *Buckholz*, of Erfurt, ufes the following method, founded upon experiments.

Take of finely pulverifed cryftals of tartar 6lb. and after boiling them in a proportionate quantity of fpring water, add by degrees, continually ftirring the mixture, pure, finely pulverifed, and well wafhed chalk, till no effervefcence be perceived, and till it have no effect on blue paper. Twenty-six ounces of chalk will be required on an average for 6 lb. of crystal of tartar. Pour off cautiously the salt solution, which contains tartarus tartarisatus, and wash the remaining tartareous selenites till it become quite free from salt and other heterogenous particles. Mix 1lb. of these selenites with 6 oz. 7 dr. and 50 gr. of white pure sulphuric acid, 1,840 specific weight, which has been diluted before with 6 or 8 times its weight of pure water. Let the mixture now be digested for three days, frequently shaking it. After this, let the tartareous acid be strained through a clean bag, and free it from selenites by expression and washing with cold water. Let all the liquors evaporate by a gentle fire. After being cool, the acid is to be dissolved in a little water, and the solution filtered, by which means a considerable quantity of selenites is separated, which is washed till it becomes insipid. The filtered liquors are then altogether evaporated to the consistence of a thin syrup, whereby again a small portion of selenites separates, which must be purified from the acid by filtering and expression. All the acid liquor is now exposed in a moderate warm place to a slow and quiet evaporation. By this mode Mr. *B.* obtained the finest white crystals. The liquor remaining over them must be poured off, and the crystals put upon paper, in which there is no glue, till they become dry. This last liquor, which is a little yellowish, is either gently evaporated, dry tartareous acid is got, or some nitric acid is added to it, till it be discoloured.

From the above quantity Mr. *B.* obtained 35—36 ounces of tartareous acid, 2 or 3 ounces included, which were absorbed by the paper. The acid prepared in this way was quite free from sulphuric acid. (*Tromsdorf's Journal of Pharmacy, No. I. in German.*)

MEDICAL

